# 12 × 6 Gy stereotactic radiotherapy for lung tumors. Is there a difference in response between lung metastases and primary bronchial carcinoma?

**DOI:** 10.1007/s00066-021-01811-3

**Published:** 2021-07-13

**Authors:** Dorota Lubgan, Sabine Semrau, Ulrike Lambrecht, Udo S. Gaipl, Rainer Fietkau

**Affiliations:** Department of Radiation Oncology, Erlangen University Hospital, Universitätsstraße 29, 91054 Erlangen, Germany

**Keywords:** Pulmonary lesion, Primary lung cancer, Stereotactic irradiation, Prognostic factor, Progression-free survival

## Abstract

**Purpose:**

The aim of this study was to evaluate the safety and long-term tumor control after stereotactic radiotherapy (SRT) with 12 × 6 Gy of patients with primary bronchial carcinoma (BC) or with pulmonary metastases (MET) of various solid tumors. Local progression-free survival (LPFS), progression-free survival (PFS), overall survival (OS), and prognostic factors were compared.

**Methods:**

Between May 2012 and January 2020, 168 patients with 206 pulmonary lesions (170 MET and 36 primary BC) were treated with 12 × 6 Gy (BED_10_ 116 Gy). The irradiated pulmonary MET were from the following cancers: 47 (27.6%) head and neck, 37 (21.8%) rectum or colon, 30 (17.6%) bronchial, 13 (7.6%) malignant melanoma, 9 (5.3%) esophageal, 9 (5.3%) sarcoma, and 25 (14.8%) other.

**Results:**

The median follow-up was 16.26 months (range: 0.46–89.34) for BC and 19.18 months (0.89–91.11) for MET. Survival rates at 3 years were: OS 43% for BC and 35% for MET; LPFS BC 96% and MET 85%; PFS BC 35% and MET 29%. The most frequently observed grade 3 adverse events (AEs) were pneumonitis (5.9% BC, 4.8% MET), pulmonary fibrosis (2.9% BC, 4% MET), and pulmonary embolism (2.9% BC, 0.8% MET). The favorable prognostic effects on overall survival of patients with MET were female gender (log-rank: *p* < 0.001), no systemic progression (log-rank; *p* = 0.048, multivariate COX regression *p* = 0.039), and malignant melanoma histology (log-rank; *p* = 0.015, multivariate COX regression *p* = 0.020). For patients with BC, it was tumor location within the lower lobe (vs. upper lobe, log-rank *p* = 0.027). LPFS of patients with metastatic disease was beneficially influenced by female gender (log-rank: *p* = 0.049).

**Conclusion:**

The treatment concept of 12 × 6 Gy is associated with 96% local progression-free survival for BC and 85% for pulmonary metastases after 3 years. There was no difference in response after SRT of primary lung carcinoma or pulmonary metastases.

**Supplementary Information:**

The online version of this article (10.1007/s00066-021-01811-3) contains supplementary material, which is available to authorized users.

## Introduction

Lung cancer is one of the most common cancers worldwide. The lungs are the second most frequent site of metastasis of various types of solid cancer. Lung metastases are frequent in head and neck cancer, gastrointestinal tumors, malignant melanoma, renal carcinomas, different types of sarcomas, and bronchial carcinoma (BC) itself [1]. Surgery is the standard for treatment of medically operable early-stage bronchial carcinoma [2] and oligometastatic pulmonary metastases [3]. Stereotactic radiotherapy (SRT) has become the preferred treatment option for medically inoperable bronchial carcinoma patients [4] with significant comorbidities, or for patients who decline surgery [5]. Locally advanced non-small-cell lung cancer (NSCLC) remains a challenging disease with persistently poor outcomes. Standard treatment for metastatic disease (MET) has historically been systemic chemotherapy. Evidence drawn from retrospective series have documented how patients with a limited pattern of disease showed improved long-term outcomes when submitted to local aggressive treatment [6]. Stereotactic radiotherapy delivers rapid and non-invasive high doses to target tumor with sparing of surrounding normal tissues. This seems to provide good results in terms of control of disease [7]. Successful local therapy for pulmonary lesions may lead to longer survival. Recent studies performing SRT of lung metastases showed high rates of local control and a low incidence of severe (grade 3–5) toxicities [8].

However, the effectiveness of SRT seems to depend not only on the irradiation protocols, but also on the pattern of development of the tumor state or distinct prognostic factors. The aim of this study was therefore to evaluate the safety and long-term tumor control of the Erlangen stereotactic radiotherapy concept with 12 × 6 Gy for patients with primary bronchial carcinoma (BC) or pulmonary metastases. We have analyzed the severity of acute and chronic adverse events, overall survival, and progression-free survival (local at irradiated lesion and systemic), and identified prognostic factors. This study differs from many other studies in the aspect that the patients with primary therapy of primary bronchial carcinoma and patients with lung metastases of various solid tumors were evaluated separately and compared to each other.

## Materials and methods

### Study design

#### Patient selection

**Fig. 1:**
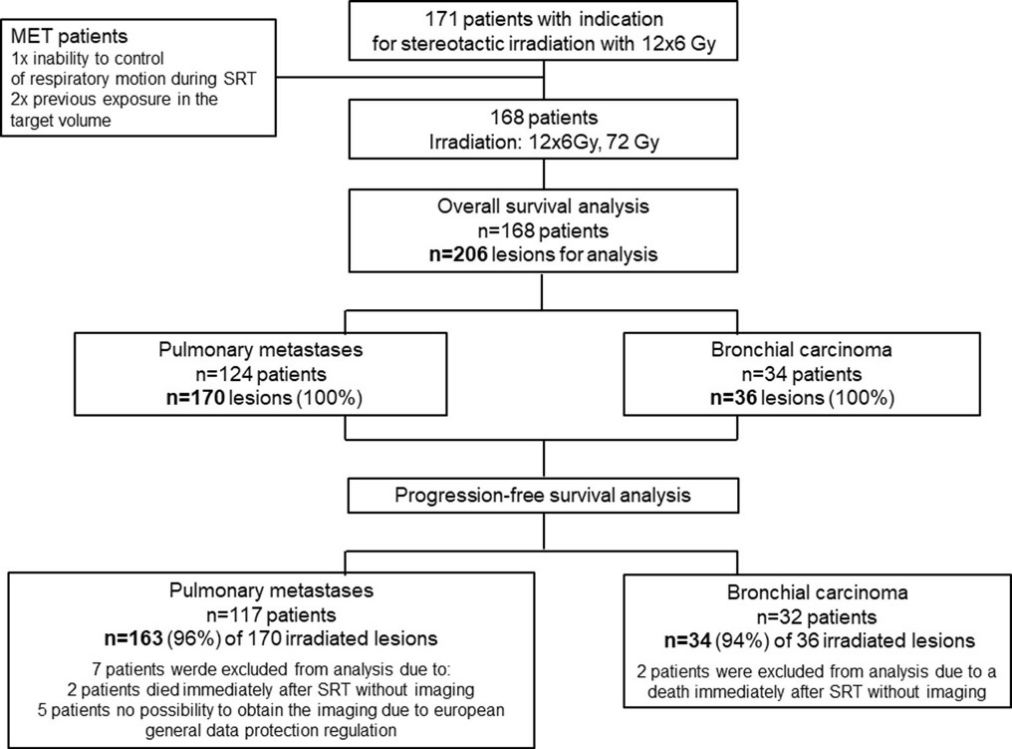
Flowchart of the study

Of 171 intended patients, 168 received 100% of the planed irradiation dose (72 Gy). For three patients with metastatic disease, we could not apply the planned dose from the beginning for reasons of radiation protection. This study was analyzed in a retrospective setting; therefore, we decided to regard the 168 patients/206 lesions as 100% (Fig. 1).

The analysis was performed by defining two efficacy subsets related to the irradiated metastases (124 patients with 170 metastases) or primary bronchial carcinoma (34 patients with 36 primary lesions). The patients were treated between May 2012 and January 2020 at the Department of Radiation Oncology of the university hospital by stereotactic irradiation. The study was conducted in accordance with the current version of the Declaration of Helsinki [9] and according to the Good Clinical Practice [10].

#### Treatment planning

SRT was performed using the dedicated stereotactic radiosurgery systems Novalis™ (BrainLAB, Feldkirchen, Germany) and Vero™ (BrainLAB). The dose was prescribed according to ICRU guidelines. A total dose of 72 Gy in 12 fractions of 6 Gy was given daily on consecutive workdays. GTV (gross tumor volume) was defined as the enhancing tumor mass (GTVUnion) based on a respiration-correlated computer tomography (CT) study. PTV (planning target volume) as the GTVUnion with a 5-mm margin in left–right and anteroposterior directions and a 7–mm margin in the craniocaudal direction. The dose was prescribed on an isodose surface (80%) that encompassed at least 95% of the PTV. The maximum dose within the PTV on a small volume of max 1 ccm was approximately 125% of the prescribed dose. Organs at risk, i.e., esophagus, heart, and spinal cord, were contoured according to the CT imaging. The treatment was conducted image guided at deep expiration with breathing commands. CT scans were performed immediately prior to STR and on days 1, 3, and 8 at maximal inspiration, maximal expiration, or during breathing baseline. The target volume was adapted in case of deviations from planning CT.

### Outcome measurements and statistical analysis

Progression-free survival (local at irradiated lesion and systemic) was assessed through repeated CT, sometimes with additional positron-emission tomography (PET-CT) and magnetic resonance imaging (MRI), according to the standard of care. Local progression-free survival (LPFS) was evaluated using the RECIST criteria as previously described [11]. The study used the International Common Terminology Criteria for Adverse Events (CTCAE) version 4.0 for toxicity and adverse events reporting. A specially designed standardized questionnaire was used by the physician to collect the toxicity data during the clinical visits at the time of irradiation and at follow-up.

Continuous variables were evaluated using descriptive statistics. Unless indicated otherwise, results are presented as mean ± SD and/or median with ranges. Standard summary statistics and two-tailed 95% confidence intervals (CI) were calculated as appropriate. All statistical analyses were performed using the Statistical Package for the Social Sciences (SPSS) version 24 (IBM Corp., Armonk, NY, USA). The level of significance for all analyses was set at α = 0.05 (two-tailed). For identification of the prognostic factors after SRT, the patient data were analyzed according to age, sex, number and location of irradiated lesions in the lung, histology, presence of other distant metastases at the irradiation timepoint, application of a concurrent systemic therapy regimen, PET-CT diagnostics prior to irradiation, and the presence of local or distant progression. The strength of the association between two variables was estimated according to Pearson [12]. Local progression-free survival (LPFS), progression-free survival (PFS), and overall survival (OS) were estimated with the Kaplan–Maier method [13], compared with the log-rank test [14], and modelled with the Cox hazard method (uni- and multivariate) [15]. Variables for which *p* < 0.3 in univariate analysis were selected for multivariate logistic regression. The date of progression was selected as the date of the first event, including local progression (a 20-percent increase in size determined by CT imaging compared to size at the end of SRT), or newly diagnosed distant metastases (from the beginning of stereotactic irradiation). Overall survival was defined as the time from the first day of irradiation until death due to any cause. Surviving patients were censored at date of last contact.

## Results

### Patient characteristics

The baseline characteristics (age, sex, tumor location in the lung, etc.) are listed for MET and BC in Table 1.

**Table 1:** Patient characteristics

General data/medical history	Metastases (MET)	Bronchial carcinoma (BC)
	*n* = 170 lesions, *n* = 124 patients	*n* = 36 lesions, *n* = 34 patients
*Age a beginning of RT (years)*		
Mean ± SD	65.79 ± 11.03	68.61 ± 12.52
	*p* = 0.175	
Median (range)	64.50 (33–90)	67.50 (33–89)
	*p* = 0.178	
*Sex*		
Male	113 (66.5%)	23 (63.9%)
Female	57 (33.5%)	13 (36.1%)
–	*p* = 0.766	
*Primary cancer diagnosis*		
BC (NSCLC + SCLC, primary tumor only)	Not applicable	36 (100%)
Head and neck	47 (27.6%)	–
Rectum or colon	37 (21.8%)	–
BC (metastatic disease)	30 (17.6%)	–
Malignant melanoma	13 (7.6%)	–
Esophagus	9 (5.3%)	–
Sarcoma	9 (5.3%)	–
Vagina or cervix uteri	8 (4.7%)	–
CUP	5 (2.9%)	–
Bladder	4 (2.4%)	–
Kidney	4 (2.4%)	–
Breast	3 (1.8%)	–
MPNST	1 (0.6%)	–
*Histology*		
Adeno	63 (37.1%)	24 (66.7%)
Squamous	77 (45.3%)	5 (13.9%)
SCLC	4 (2.4)	4 (11.1%)
BC (no histology or unspecified)	1 (0.6%)	3 (8.3%)
Malignant melanoma	13 (7.6%)	–
Sarcoma	9 (5.3%)	–
Kidney spindle cell	2 (1.2%)	–
MPNST spindle cell	1 (0.6%)	–
*Location in the lung*		
Upper (right upper + middle and left)	101 (59.4%)	23 (63.9%)
Lower (right and left)	69 (40.6%)	13 (36.1%)
*Number of irradiated lung lesions/patient*		
1	90 (52.9%)	32 (88.9%)
2	54 (31.8%)	4 (10.8%)
3	18 (10.6%)	–
4	8 (4.7%)	–
*Lung metastases*		
Metachronous	163 (95.9%)	Not applicable
Synchronous	7 (4.1%)	–
*Interval from primary cancer diagnosis to irradiation of metastasis (months)*		
Mean ± SD	39.14 ± 38.92	Not applicable
Median (range)	25.28 (2.0–225)	–
*PET-CT prior to irradiation*		
No	140 (82.4%)	18 (50.0%)
Yes	30 (17.6%)	18 (50.0%)
*Other distant metastases at irradiation time point*		
Yes	101 (59.4%)	16 (44.4%)
No	69 (40.6%)	20 (55.6%)
*Concurrent therapy during irradiation*		
No	136 (80.0%)	22 (61.1%)
Antibody	20 (11.8%)	1 (2.8%)
Chemotherapy	7 (4.1%)	12 (33.3%)
Tyrosine kinase inhibitor	3 (1.8%)	–
Chemotherapy + antibody	3 (1.8%)	1 (2.8%)
Tyrosine kinase + PARP inhibitor	1 (0.6%)	–
*Irradiation*		
12 × 6 Gy	170 (100%)	36 (100%)
*PTV total dose (Gy)*		
Mean ± SD	73.01 ± 1.55	73.00 ± 1.46
	*p* = 0.985	
Median (range)	72.60 (70.27–76.67)	72.81 (71.05–75.27)
	*p* = 0.798	
BED (α/β = 10)	116.16	116.5
EQD2	96.8	97.08
*Follow-up time (months)*		
Mean ± SD	22.96 ± 19.86	26.45 ± 20.65
	*p* = 0.339	
Median (range)	16.26 (0.46–89.34)	19.18 (0.89–91.11)
	*p* = 0.199	
*Tumor stage for BC*		
cT1	–	15 (41.7%)
cT2	–	12 (33.3%)
cT3	–	5 (13.9%)
cT4	–	2 (5.6%)
Missing in the database	–	2 (5.6%)
*Nodal status for BC*		
cN0	–	22 (61.1%)
cN+	–	12 (33.3%)
Missing in the database	–	2 (5.6%)

Data are number of lesions (%) unless otherwise stated. *P*-value: analysis of covariance: Mann–Whitney *U *(median) and Student’s t (mean) test, χ^2^ test in case of categorial data

MPNST malignant peripheral nerve sheath tumor

**Fig. 2:**
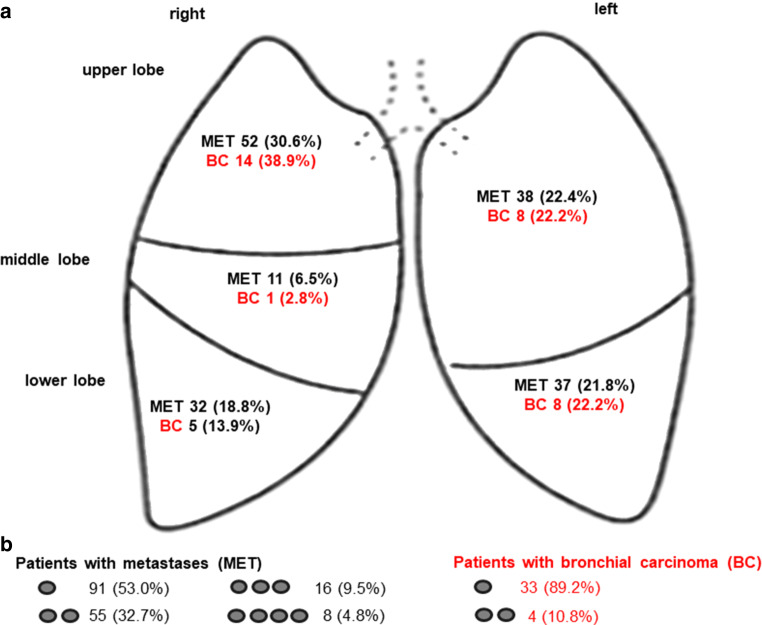
**a** Allocation of the lesions to the area of the lung for patients with metastases (*black*) and with bronchial carcinoma (*red*). **b **Number of irradiated lung lesions per patient

The most common primary tumor site for MET patients was head and neck (27.6%, 47 cases), followed by rectum or colon (21.8%, 37 cases). Primary bronchial carcinomas were almost always adenocarcinoma (66.7%, 24 cases), followed by squamous cell carcinoma (13.9%, 5 cases). For patients with metastatic disease the histology was predominantly squamous carcinoma (45.3%, 77 cases) or adenocarcinoma (37.1%, 63 cases). Pulmonary lesions occurred most frequently in the right upper lobe: 30.6% for metastatic disease vs. 38.9% for BC. The majority of patients had one or two lesions irradiated with 12 × 6Gy (maximally four, illustrated in Fig. 2).

Tumor stages for primary bronchial carcinoma were: ≤ cT1 (41.7%, 15) and ≥ cT2 (52.8%, 19). Irradiation was delivered to the PTV in the MET study group to a median total dose of 72.60 Gy (range: 70.27–76.67 Gy) and of 72.81 Gy (range: 71.05–75.27 Gy) in the BC group. The median calculated biological effective dose (BED) was 116.16 Gy for MET and 116.5 Gy for BC patients based on α/β = 10. The patients with lymph node or other extrathoracic metastases were sequentially treated with chemoradiation (e.g., mediastinal radiotherapy with boost up to 66 Gy in case of N +) according to the standard of care. Systemic therapy of various regimens was administered concurrently (4 weeks prior and during) with irradiation for 20% of patients with metastases and 38.9% of patients with BC.

### Survival outcomes and prognostic factors

The median follow-up at the time of overall survival analysis was 16.26 months (range: 0.46–89.34) for MET and 19.18 months (range: 0.89–91.11) for BC patients. We analyzed the imaging in 163 (96%) of 170 irradiated cases for MET patients and in 34 (94%) of 36 cases for BC. In a total of 9 cases, the imaging datasets were not available for analyses (Fig. 1).

#### Local progression-free survival (LPFS)

**Fig. 3:**
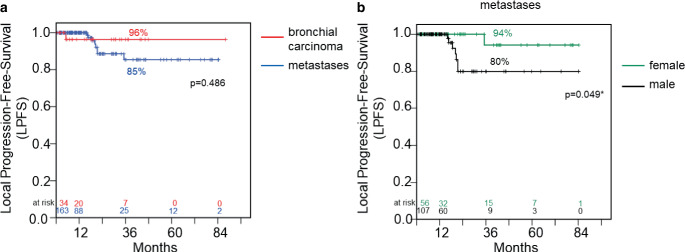
**a** Local progression-free survival rates of patients with metastases (*blue curve*) in relation to patients with primary bronchial carcinoma (*red curve*) after irradiation. **b** Local progression-free survival rates of patients with metastases: female gender (*green curve*) in relation to male gender (*black curve*) after irradiation. Survival rates are given in % for 3‑year survival (36 months). Significant coherencies (*p* < 0.05, log-rank test) are marked with an *asterisk*

LPFS of 96% for patients with bronchial carcinoma differs not significantly from 85% for patients with metastases after 3 years (Fig. 3a and Table 2). Detailed information including dose distribution for patients with local progression is presented in Table 3.

**Table 2:** Log-rank survival analysis for patients with metastases compared to patients with primary bronchial carcinoma calculated at 3 years after therapy and univariate analysis according to Cox regression. Significant coherencies (*p* < 0.05) are marked with an asterisk

	Log-rank		Univariate analysis		
	Survival rate at 3 years	*p*-value	HR	95% CI	*p*-value
*Overall survival*		0.823	1.056	0.656–1.700	0.824
MET	35%				
BC	43%				
*Local progression-free-survival*		0.486	0.485	0.064–3.885	0.496
MET	85%				
BC	96%				
*Progression-free-survival*		0.438	0.804	0.462–1.398	0.439
MET	29%				
BC	35%

**Table 3:** Characteristics of patients with local progression

**Patient no.**	Age at beginning of RT (years)	Sex	Primary tumor site	Number of irradiated pulmonary lesions	Number of progressive pulmonary lesions	Histology	Location in the lung	Concurrent therapy during irradiation	Follow-up time (months)	Total dose (Gy)
*Pulmonary metastases (all metachronous)*										
1	65	Male	Rectum	2	2	Adeno	Right middle Left upper	No	16.03 16.69	72.40 72.00
2	65	Male	Lung	2	1	Squamous	Right lower	No	20.69	72.32
3	70	Female	Malignant melanoma	2	1	Malignant melanoma	Right upper	No	33.15	68.30
4	73	Male	Colon	1	1	Adeno	Left lower	No	18.62	72.20
5	57	Male	Esophagus	3	3	Squamous	Right lower Left lower Left lower	No	20.56 21.41 21.41	71.86 72.21 72.55
										**Median** **72.20**
*Primary bronchial carcinoma*										
6	85	Female	Lung	1	1	Adeno	Right upper	No	5.08	71.44
–	–	–	–	–	**Total 9**	–	–	–	–	–

A highly relevant beneficial prognostic factor for LPFS (log-rank: *p* = 0.049, univariate COX regression analysis: *p* = 0.036) in patients with metastases was female gender (Fig. 3b). The LPFS rate was for females 94% after 3 years compared to 80% for male patients. In bronchial carcinoma patients the gender had no impact on LPFS (log rank: *p* = 0.228).

#### Progression-free survival (PFS)

The PFS rate at 3 years for patients with lung metastases was 29% compared to 35% for patients with bronchial carcinoma (Online Resource 1). The difference was not significant (log-rank: *p* = 0.438, univariate COX regression 0.439, Table 2).

#### Overall survival (OS)

**Fig. 4:**
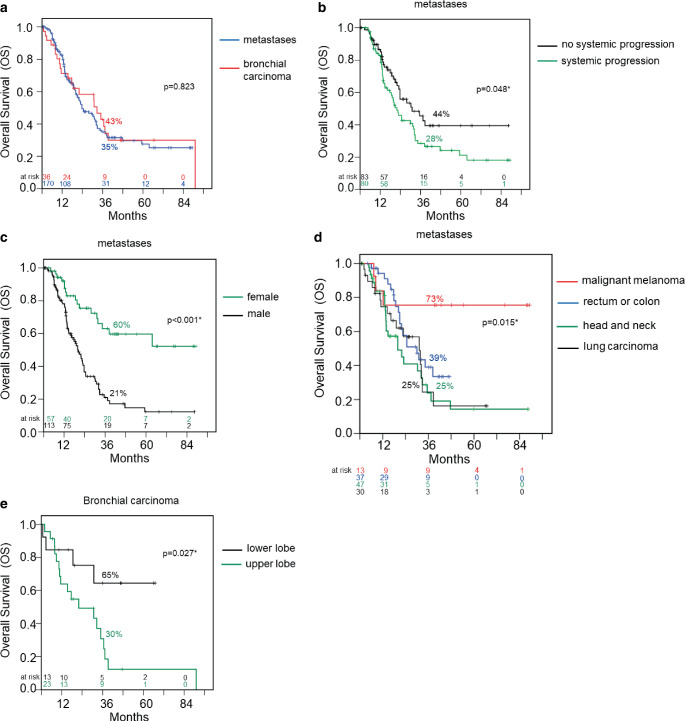
**a** Overall survival rates of patients with metastases (*blue curve*) in relation to patients with primary bronchial carcinoma (*red curve*) after irradiation. **b** Overall survival rates of patients with metastases. Impact of systemic progression: patients with no systemic progression (*black curve*) in relation to systemic progression (*green curve*) after irradiation. **c** Overall survival rates of patients with metastases. Gender: female (*green curve*) in relation to male (*black curve*) after irradiation. **d** Overall survival rates of patients with metastases. Primary tumor site: malignant melanoma (red curve) in relation to rectum or colon (*blue curve*), head and neck (*green curve*), and lung carcinoma (*black curve*) after irradiation. **e** Overall survival rates of patients with bronchial carcinoma. Tumor location: lower lobe (*black curve*) in relation to upper lobe (*green curve*) after irradiation. Survival rates are given in % for 3‑year survival (36 months). Significant coherencies (*p* < 0.05, log-rank test) were marked with an *asterisk*

Overall survival at 3 years was 35% for MET und 43% for BC patients (Fig. 4a). There was no statistically significant difference between the groups (Table 2). The non-tumor-related causes of death for 7 patients with metastatic disease were as follows: poor general condition (2), diabetic coma, bronchopulmonary infection, infection of port catheter, cardiac insufficiency, renal insufficiency; and for two bronchial carcinoma patients: poor general condition and chronic obstructive pulmonary disease. The most common cause of mortality was a systemic progression in both groups.

The presence of systemic progression was a highly relevant prognostic factor for overall survival in the MET group. The Kaplan–Meier analyses showed that the MET patients with no systemic progression had a significantly better (*p* = 0.048) overall survival of 44% after 3 years compared to patients with systemic progression (OS 28%, Fig. 4b). The multivariate analysis (*p* = 0.039, Table 4) confirms this statement. For BC patients the difference (60% versus 31%, diagram not shown) after 3 years was not statistically significant (log-rank *p* = 0.064, univariate Cox *p* = 0.072, Table 4).

**Table 4:** Univariate (MET and BC) and multivariate analysis (MET only) of the influence of patient- and therapy-related parameters to overall survival for patients treated with 12 × 6 Gy. Significant coherencies (*p* < 0.05) were marked with an asterisk. Hazard ratios and 95% confidence interval

		Metastases						Bronchial carcinoma		
		Univariate			Multivariate			Univariate		
Variables	Category	HR	95% CI	*p*-value	HR	95% CI	*p*-value	HR	95% CI	p‑value
Sex	Female vs. male	0.311	0.182–0.531	< 0.001*	0.529	0.232–1.208	0.131	0.560	0.217–1.448	0.232
Systemic progression	Yes vs. no	0.647	0.418–1.001	0.051*	0.423	0.188–0.956	0.039*	0.425	0.167–1.081	0.072
Primary tumor site	Malignant melanoma vs. other	0.224	0.069–0.723	0.012*	0.009	0.000–0.478	0.020*	Not applicable		
Histology	Adeno vs. squamous	0.688	0.435–1.088	0.110	0.816	0.343–1.943	0.646	1.954	0.438–8.714	0.380
Age at beginning of RT	Years	1.011	0.990–1.031	0.308	Not possible			1.002	0.966–1.039	0.923
Location in the lung	Upper vs. lower	1.467	0.964–2.231	0.074	1.087	0.566–2.089	0.802	0.310	0.103–0.929	0.036*
Number of irradiated lung lesions/patient	1 + 2 vs. 3 + 4	0.777	0.457–1.321	0.352	Not possible			Not possible		
Other distant metastases at irradiation timepoint	Yes vs. no	1.220	0.802–1.858	0.353	Not possible			0.530	0.637–3.675	0.342
Concurrent therapy during irradiation	Yes vs. no	1.115	0.637–1.950	0.704	Not possible			0.498	0.200–1.243	0.135
PET-CT prior to irradiation	Yes vs. no	0.976	0.527‑1.809	0.940	Not possible			1.476	0.610–3.570	0.388
Local progression	Yes vs. no	0.800	0.367‑1.743	0.547	Not possible			0.169	0.20–1.452	0.105
Tumor stage for BC	T1 vs. T2-T4	Not applicable						0.396	0.150–1.045	0.061
Nodal status for BC	N0 vs. N +	Not applicable						1.859	0.723–4.780	0.198

Female gender has a favorable impact on OS for patients with metastases. The OS rate of 60% for female patients was better than 21% for male patients (Fig. 4c). The difference was statistically significant in Kaplan–Meier and univariate analysis (*p* < 0.001), but not in multivariate analysis (*p* = 0.131, Table 4).

For bronchial carcinoma patients the difference was not significant (57% female vs. 30% male, log-rank: OS *p* = 0.225, diagram not shown; and univariate analysis *p* = 0.232, Table 4).

Furthermore, a significantly better overall survival of 73% (*p* = 0.015) at 3 years is observed if metastases originated from malignant melanoma compared to other tumor types (up to 40%, Fig. 4d). The univariate Cox (*p* = 0.012) and multivariate Cox analyses (*p* = 0.020) show the same results (Table 4).

The “tumor location” variable showed a benefit in OS in the BC group. We compared the tumor location within the upper (right and left) including middle lobe to a location within the lower lobe (right and left). After 3 years the overall survival rate of 65% for patients with a “lower lobe” location was significantly better (log-rank: *p* = 0.027, univariate COX analysis *p* = 0.036) compared to 30% for an “upper lobe” location (Fig. 4e; Table 4).

The group of patients with metastatic disease showed a contrasting pattern. Patients with lesions within the upper lobe showed an overall survival of 40% compared to the patients with a tumor location in the lower lobe at 26% (diagram not shown). The difference was not significant (*p* = 0.071 log rank, *p* = 0.074 univariate analysis).

For bronchial carcinoma patients, tumor stage ≤ T1 show a trend (*p* = 0.053) for better overall survival at 3 years (57% ≤ T1 compared to 35% ≥ T2; Online Resource 2; Table 4).

### Adverse events (AE)

The overall incidence of AEs suspected to be related to the irradiation was low. There were no interruptions of irradiation due to toxicity. No deaths occurred during irradiation or due to irradiation. Four patients mentioned in the Survival outcomes and prognostic factors section (as without imaging) died immediately after irradiation due to primary head and neck tumor bleeding (one MET patient), toxicity in consequence of concurrent chemotherapy (one BC patient), and serious infection (one BC und one MET patient). No heart toxicity, lung bleeding, esophagitis, or dysphagia were observed. The most frequently observed grade 3 AE was pneumonitis for both groups (5.9% BC, 4.8% MET), followed by pulmonary fibrosis (2.9% BC, 4% MET) and pulmonary embolism (2.9% BC, 0.8% MET). We observed five rib fractures in 3 patients (8.8%) with BC and 2 patients (1.6%) w metastatic disease.

## Discussion

This study compared the safety and long-term survival outcomes after a stereotactic radiotherapy with 12 × 6 Gy for patients with primary bronchial carcinoma and patients with lung metastases of various solid tumors. We have identified prognostic factors that may influence local progression-free-survival (LPFS) and overall survival.

From our results, female gender, absence of systemic progression, and malignant melanoma histology for MET patients, and tumor location within the lower lobe or tumor stage ≥ T1 for BC patients were favorable prognostic factors.

The major efficacy outcome measure of the trial was local progression-free survival. 3‑year outcome data showed 96% LPFS for lung carcinoma patients and 83% for MET patients. Our LPFS rate of 83% for patients with lung metastases was comparable with 81.3% (3 years) in one of the largest multicenter (68 centers) analyses consisting of 1547 cases by Yamamoto et al. [16], and lower compared to 94.1% or 89% as seen in retrospective series consisting of 129 cases by Borm et al. [8] or 61 cases by Ricardi et al. [17], respectively. The treatment schedules contained 3–7 fractions up to a biologically equivalent dose (BED_10_) of 75.0–289.5 Gy (median 105.6 Gy) [16]. The patients in the analysis of Borm et al. and Ricardi et al. were treated with a total dose that ranged between 14 and 45 Gy, given mainly in 3 or 5 fractions [8], or 1–4 fractions [17]. Our local failure rate for BC patients of 4% after 3 years was considerably lower compared to 2‑year rates of 38.6% or 29% reported by Bradley et al. [18] and McDermott et al. [19], respectively.

With the excellent local control, no uniform pattern of failure is clearly identifiable in our study. In 8 of 163 (5%) of MET and in one (3%) of 34 of BC lesions, we observed local progression. Various dose fractionation schedules are reported for lung lesions in the literature, varying between 1 and 10 fractions; however, the optimal dose remains unknown and a comparison of outcomes challenging. The schedules with a biologically equivalent dose (BED_10_) of 100 Gy or more are associated with high local disease control rates [20–22]. We recorded no difference in radiotherapy delivery between the investigated groups. Except for the above mentioned malignant melanoma patient, we administered the recommended dose (in median of 72.20 Gy, BED_10_ 115.52) to the target volume, even for patients with local progression.

Female gender was the only independently significant highly beneficial prognostic factor for LPFS of patients with metastatic disease, but not for bronchial carcinoma. Klement et al. evaluated a radiobiological model for long-term prediction of tumor control probability (TCP) for stereotactic irradiation of pulmonary metastases. The female sex was associated with higher control probability [23]. Rodrigues et al. reported in contrast that LPFS was negatively influenced by female gender after SBRT for early-stage inoperable primary lung tumors [24]. No other factors were found to have an impact on local LPFS, probably due to, fortunately, very high response rates.

Much effort has been focused on the ability of SRT to control tumors locally, but the risk of distant metastasis remains a major issue for both patient groups. During follow-up, the life expectancy of the patients in the herein presented study was predominantly limited by systemic progression. A PFS rate of 29% at 3 years for patients with lung metastases differs non-significantly from 35% for patients with bronchial carcinoma. In terms of the incidence of metastases in BC, our results appear to be quite different to results of the RTOG 0236 trial, with 48% disease-free survival 3 years after irradiation [25], or an analysis of Abdalmassih et al. (2020) with 51% at 2 years [26]. It is generally difficult to compare progression-free survival due to heterogeneous inclusion criteria, especially the number of other distant metastases and variety of irradiation treatments. In our series we did not recognize any independent prognostic factors for PFS.

Successful local therapy for lung lesions, regardless of whether primary or metastatic, may lead to longer survival. We observed no difference in overall survival after 3 years between the groups (35% for MET vs. 43% for BC). The OS rate of 35% for patients with irradiated metastases was lower in our study than the average values (range 38–84.3%) of previous studies [8, 17, 27–31]. We agree here with the opinion of previous reports that the careful selection of patients (e.g., no other extrathoracic metastases) for analysis [17, 27, 32] positively influenced the overall survival. Clinical outcomes after SRT for inoperable NSCLC vary significantly between different studies: 3‑year overall survival (OS) ranges from 37% to 72% [25, 33, 34]. In our series, the OS rate for patients with primary BC after 3 years was 43%. Only a minority of reports on SRT have a long follow-up [6]. Therefore, these results should be interpreted with caution, since SRT is currently still reserved for patients unfit or unwilling to undergo surgical treatment and this might have a considerable impact on the survival rates.

Previous studies confirmed that no new metastases during follow-up [28] and a long event-free interval [35, 36] are beneficial prognostic factors for overall survival of patients with metastases. MET patients in our study with “no systemic progression” have a significantly better overall survival of 44% after 3 years compared to patients with systemic progression (OS 28%). For BC patients we observed no difference.

A highly beneficial prognostic factor for patients with metastases but not for patients with primary lung cancer was female gender (MET 3‑year OS: 60% vs. 21%, *p* < 0.001). De Vin et al. showed the same scope [37]. Little is known about the impact of gender for MET patients after SRT. On the other hand, a retrospective analysis of 243 patients from the Japanese Lung Cancer Registry suggested that gender is one of the independent prognostic factors for lung carcinoma [38, 39]. For early-stage inoperable NSCLC, when compared to men 2 years after SRT, women had a statistically superior OS of 75% compared to 52% for men [26]. In a patient cohort with limited-disease SCLC, female gender was significantly associated with longer OS [38, 40].

Various types of cancer show significant differences in tumor response after SRT. Some authors described that better local control was observed if metastases did not originate from the colon [41–44] and local failure of irradiated metastases of colon carcinoma has been reported to have a correlation with worse overall survival [45]. Our analysis regarding the impact of local control could not confirm this statement, but when comparing our results for metastases from various tumors, we found that malignant melanoma histology had a highly relevant impact on overall survival against other tumor types. Malignant melanoma patients responded better (OS 73%) compared to other tumor types (OS 25–39%) after 3 years.

We asked ourselves: Why was the overall survival of malignant melanoma patients better compared to patients with other histologies? Additional administration of pembrolizumab had an impact on overall survival in our analysis. We irradiated 13 lesions from 8 patients. One patient had local progression (Table 2). Almost all of our malignant melanoma patients were treated successfully with pembrolizumab after irradiation (but not prior to) for a longer period of time. Only one female patient did not respond and died. Since the publication of the KEYNOTE-001 study, it has been known that pembrolizumab demonstrates robust antitumor activity [46].

The factor “tumor location in the lung” was a highly relevant (*p* = 0.027 log-rank, Cox: univariate analysis *p* = 0.036) prognostic factor for OS in patients with primary lung cancer. Patients with lesions within the lower lobe responded better (OS 65% after 3 years) compared to patients with lesions in the upper lobe (OS 30%). For patients with metastases, it is exactly the opposite, but non-significant. In the literature review of 20 publications reporting outcomes for lung tumors treated with stereotactic radiotherapy, tumor location in the lung did not appear to impact OS, but did impact toxicity [5]. As might be expected, grade 3 and 4 toxicities were more prevalent for central tumors [5, 47]. With respect to our small sample size and heterogenous patient population including N + and M1 patients, we found that overall survival of bronchial carcinoma patients was influenced by tumor stage. The patients with ≥ T1 tumors (OS 57%) showed a trend toward better overall survival than the patients with ≥ T2 tumors (OS 35%). Rodrigues at al. showed that T2 tumors were associated with lower local control rates than T1, with no significant impact on overall survival [24]. Other factors that were evaluated during statistical analysis, included age, number of irradiated lesions in the lung, presence of other distant metastases at the irradiation timepoint, application of a concurrent systemic therapy regimen, PET-CT diagnostics prior to irradiation, and the presence of local progression, were all found to be non-statistically significant for LPFS, PFS, and OS.

Using higher doses in stereotactic radiotherapy is a clinical decision in which tumor control is weighed against toxicity. Unfortunately, the higher biologically equivalent doses (BED) can also result in toxicity. The 12 × 6 Gy (BED_10_ 116 Gy) stereotactic radiotherapy was generally well tolerated, which is reflected in compliance. In the majority of studies that reported adverse events these were grade 1 and grade 2 toxicity, without the need for further treatments [8, 27, 31]. In our analysis, the overall incidence of AEs suspected to be related to the irradiation was low and showed no clinical correlate in most cases. Our findings are in accordance with previous studies that described high-grade (≥ 3) adverse events to be well under 10% [8, 17, 27–32, 35, 48].

The strengths of our findings are a homogeneous and safe radiotherapy concept with 12 × 6 Gy, the large series of 206 cases, and the long follow-up period. Nevertheless, identifying patients who are most likely to benefit from stereotactic therapy is probably the most significant challenge, as well as obtaining high quality prospective data. Furthermore, there is still a need for prospective studies with clearly defined inclusion criteria with a focus on the presence of extrathoracic metastases of lung lesions, including primary lung cancer and metastases of solid tumors.

## Conclusion

The treatment concept of 12 × 6 Gy might be safe and was associated with 96% local progression-free survival for BC and 85% for pulmonary metastases after 3 years. The most common cause of failure was systemic progression in both groups. Primary bronchial carcinoma responded similarly to lung metastases of various solid tumors.

## Supplementary Information

**Online Resource 1 **Progression-free-survival rates (distant metastases) of patients with metastases (*blue curve*) in relation to patients with primary bronchial carcinoma (*red curve*) after irradiation.

**Online Resource 2 **Overall survival rates of patients with bronchial carcinoma. Tumor stage: ≤ T1 (*black curve*) in relation to ≥ T2 (*green curve*) after irradiation. Survival rates are given in % for 3‑year survival (36 months). Significant coherencies (*p* < 0.05, log-rank test) are marked with an *asterisk*.
