# Supplementary figures and images for: 12 × 6 Gy stereotactic radiotherapy for lung tumors. Is there a difference in response between lung metastases and primary bronchial carcinoma?
Source: Strahlenther Onkol. 2021 Jul 13;198(2):110–22. doi: 10.1007/s00066-021-01811-3 (PMC8789716; doi:10.1007/s00066-021-01811-3)

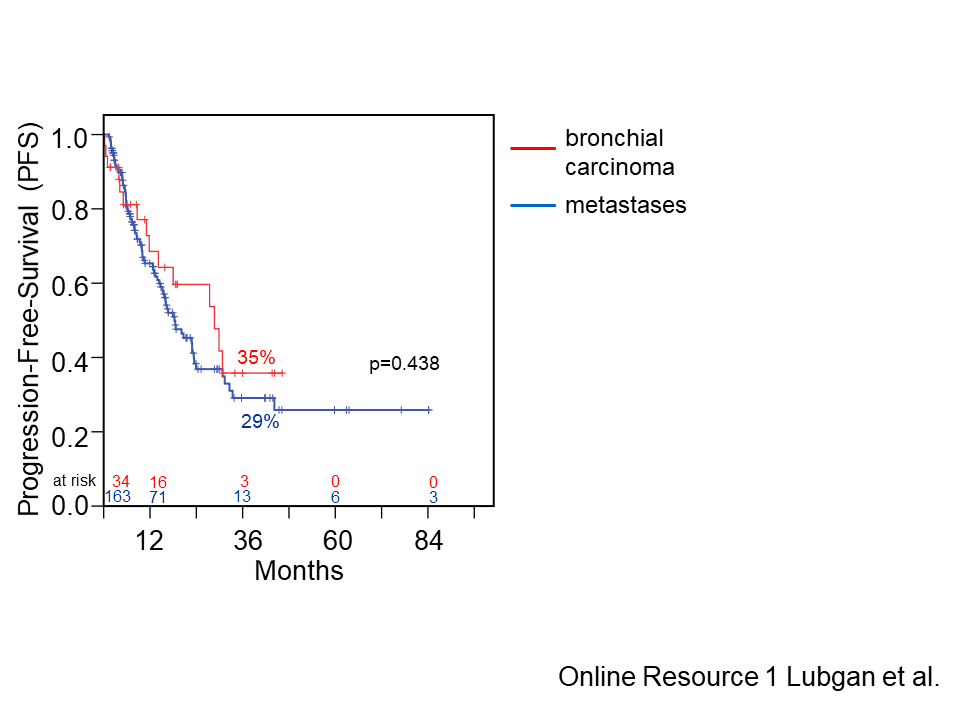

Supplement: Supplementary file 1 — Online Resource 1 Progression-free-survival rates (distant metastases) of patients with metastases (blue curve) in relation to patients with primary bronchial carcinoma (red curve) after irradiation. [file 66_2021_1811_MOESM1_ESM.tif]

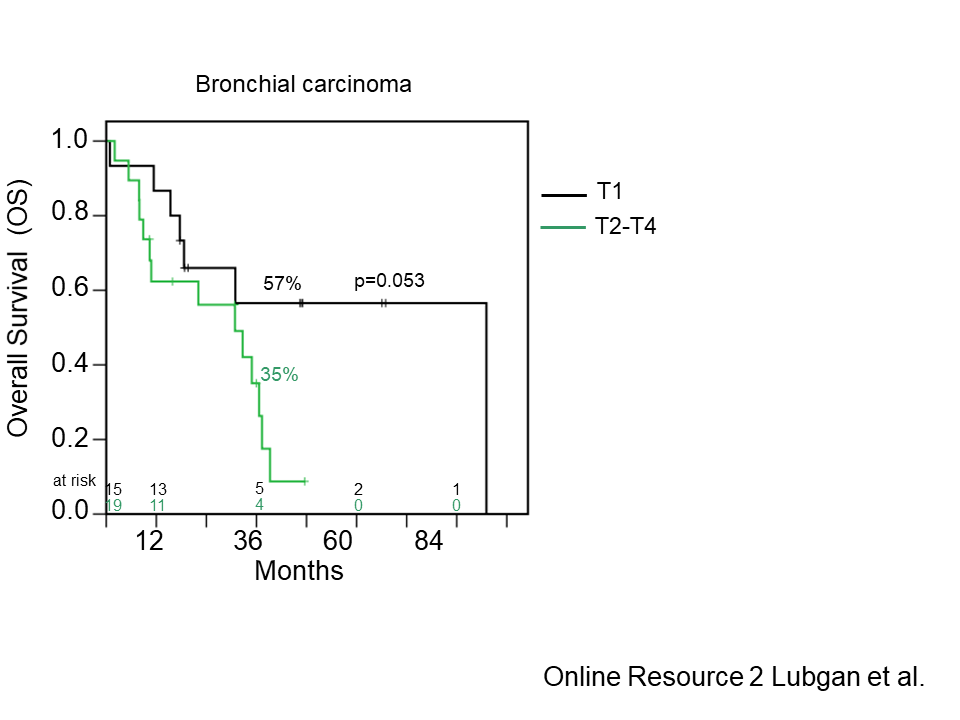

Supplement: Supplementary file 2 — Online Resource 2 Overall survival rates of patients with bronchial carcinoma. Tumor stage: ≤ T1 (black curve) in relation to ≥ T2 (green curve) after irradiation. Survival rates are given in % for 3‑year survival (36 months). Significant coherencies (p < 0.05, log-rank test) are marked with an asterisk. [file 66_2021_1811_MOESM2_ESM.tif]
